# The Choroidal Vascularity Index Versus Optical Coherence Tomography Angiography in the Evaluation of the Choroid with a Focus on Age-Related Macular Degeneration

**DOI:** 10.3390/tomography9040116

**Published:** 2023-08-04

**Authors:** Mariachiara Di Pippo, Claudia Santia, Daria Rullo, Chiara Ciancimino, Flaminia Grassi, Solmaz Abdolrahimzadeh

**Affiliations:** Ophthalmology Unit, Neurosciences, Mental Health, and Sense Organs (NESMOS) Department, Faculty of Medicine and Psychology, University of Rome Sapienza, St. Andrea Hospital, 00189 Rome, Italy; mariachiara.dipippo@uniroma1.it (M.D.P.); chiara.ciancimino@uniroma1.it (C.C.);

**Keywords:** choroidal vascularity index, optical coherence tomography angiography, spectral-domain optical coherence tomography, choroid, age-related macular degeneration, multimodal retinal imaging

## Abstract

The choroid is the most vascularized structure of the eye and it is fundamental for the trophism of the outer retina. Its proper functioning and homeostasis represent key points in maintaining normal retinal physiology. Choroidal alterations may be implicated in the development and progression of numerous pathologies; therefore, in-depth studies using imaging techniques can be of crucial relevance to understanding the pathophysiology of retinal-choroidal diseases. The advent of spectral-domain optical coherence tomography (SDOCT) has enabled the non-invasive study of the choroid in vivo and the most recent development, optical coherence tomography angiography (OCTA), allows for the high-resolution visualization of the choriocapillaris and the choroid in regard to vascularization. The choroidal vascularity index (CVI) is a new parameter calculated on SDOCT scans and is defined as the ratio of the luminal area to the total choroidal area. In this review, a study of the choroid using OCTA and CVI will be evaluated in depth and the pros and cons of these two methods will be analyzed, with a particular focus on age-related macular degeneration.

## 1. Introduction

The choroid, a highly vascularized eye structure, performs a crucial function in preserving retinal homeostasis. It plays a pivotal role in supplying oxygen and essential nutrients to the outer retinal cells and retinal pigment epithelium (RPE). Comprising blood vessels encased within stromal tissue consisting of connective tissue, melanocytes, nerves, and extracellular fluid, the choroid can be categorized into three layers: the innermost choriocapillaris (CC), Sattler’s layer, and the outermost Haller’s layer [1]. Choroidal vascular alterations are implicated in several retinal pathologies, therefore, a detailed evaluation using imaging techniques is currently crucial to the understanding of disease pathophysiology [2]. Optical coherence tomography angiography (OCTA) is a recent technique derived from developments in spectral domain optical coherence tomography (SDOCT) technology [1,3]. OCTA allows for a rapid, noninvasive, and detailed visualization of the retinal and CC vasculature. The concept behind OCTA is that of motion detection, which means that only what is moving inside the tissue is visualized and, considering that blood is the only moving element of the retina, vascular flow is detected using the movement of red blood cells as the intrinsic contrast agent [4]. A novel parameter that can be calculated using SDOCT scans is the choroidal vascularity index (CVI), which is defined as the ratio of the luminal area to the total choroidal area. Briefly, the CVI is calculated with the binarization of enhanced-depth imaging (EDI) SDOCT images [5]. In this review, OCTA and CVI will be described, evaluating their merits and limitations, and their current role in choroid dysfunction in the context of age-related macular degeneration (AMD).

## 2. Choroidal Parameters

### 2.1. Anatomy of Choroid

The choroid is the most vascularized structure of the eye; compared to other tissues in the body, it has the highest blood flow per unit weight [1]. It is the posterior portion of the uvea and the most extensive portion of the vascular tissue interposed between the deep face of the sclera and the deepest layer of the retina [6]. The thickness of the choroid is not uniform and is highly variable, depending on the blood flow of the choroidal vessels, increasing when there is a state of filling and decreasing when there is a state of relative emptying [7]. The inner face of the choroid is tightly connected to the retina; indeed, its adhesion with the outermost layer of the retina is so strong that spontaneous separation between the choroid and the retina is impossible.

The close anatomical proximity of the choroid and the retina underscores the crucial function of the choroid. It serves as a vital source for retinal nourishment and maintaining balance, ensuring a supply of oxygen and nutrients to the RPE cells and outer retinal layers while eliminating metabolites [8]. The choroid is structurally categorized into five layers, arranged from innermost to outermost: Bruch’s membrane, choriocapillaris (CC), Sattler’s layer, Haller’s layer, and the suprachoroidal space. The CC consists of a monolayer vascular layer, divided into functional units arranged in a mosaic pattern; the capillaries are small in diameter and fenestrated in the posterior pole of the retina, ensuring a high blood flow in the macular area and becoming progressively larger towards the periphery [9,10]. Sattler’s layer is composed of small-caliber arteries and veins, and Haller’s layer is constituted of large-caliber arteries and veins. The suprachoroidal space contains collagen fibers, melanocytes, and fibroblasts [11].

### 2.2. SDOCT

The introduction of SDOCT has allowed for images to be obtained with an improved axial resolution (about 3–5 μm) and higher acquisition speed (20,000–100,000 A-scans/s), with respect to previous time domain technology [12,13], allowing for the visualization of retinal structures with a histological-like resolution [14]. SDOCT is routinely used in the management of a variety of ocular diseases [12,13,15,16]. SDOCT technology, with multiple A-scans, generates images corresponding to the frontal sections of retinal layers, called “en face” scans, and transverse and sagittal sections, called “B-scans” [17,18]. Enhanced depth imaging (EDI) technology has been incorporated into the framework of some commercially available SDOCT instruments. This technology allows for a high-resolution visualization of deeper structures such as the choroid and the sclera [19]. In recent times, a new technology called swept-source OCT (SSOCT) has been introduced, utilizing a longer wavelength (1050 nm). This advancement enables enhanced signal penetration through the RPE, pigment deposits, drusen, and various other structures. Consequently, the SSOCT offers an improved visualization of the choroidal layers [20,21,22,23].

### 2.3. Choroidal Thickness

**Figure 1:**
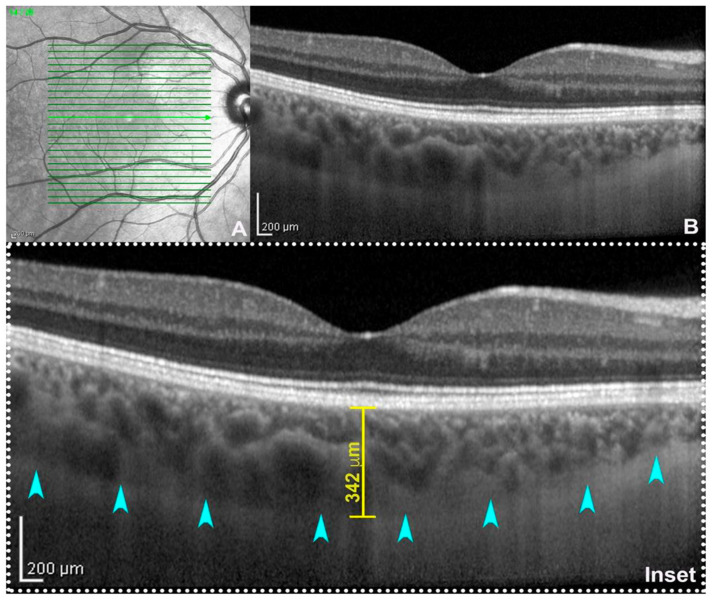
Enhanced depth imaging (EDI: (**A**) near-infrared reflectance; (**B**) spectral-domain optical coherence tomography (SD-OCT, Heidelberg Engineering, Heidelberg, Germany) subfoveal B-scan acquired using EDI-mode. On magnification (inset), the choroid–scleral junction is clearly detectable (teal arrowheads) allowing the calculation of subfoveal choroidal thickness traced between the outer border of the retinal pigment epithelium and the inner surface of the choroid–scleral junction through a digital caliper. From [33].

Choroidal thickness (CT) was one of the first parameters used to study the state of choroidal health. The first evidence dates back to the 70s, when this parameter was calculated from ultrasound images [24]. The evolution of ocular imaging technologies and the introduction of SDOCT with EDI technology has allowed for the standardization of the measuring method of CT and made it one of the main research methods in choroidal-retinal diseases [25,26,27]. CT can be calculated with manual or semi-automatic measurements on EDI scans from the inner choroidal boundary, corresponding to the RPE to the outer choroidal boundary, corresponding to the choroid–scleral junction [28,29] (Figure 1). Measurements can be performed under the fovea or at various distances from the fovea, producing even CT maps of the whole zone of interest. For example, measurements are commonly made on EDI scans with the manual caliper tool at the fovea and at variable intervals of 500 μm or 750 μm nasally and temporally, with respect to the fovea, to analyze up to 1500 or 2500 μm from the fovea [30]. This parameter has been and is now widely used in clinical practice and research to recognize, diagnose, and manage a range of ocular conditions [31]. Nevertheless, it is a parameter that is burdened by several limitations. It has a great variability and depends on numerous factors, including age, axial length, intraocular pressure, and time of day [32]. It does not provide information on other important aspects, such as vascularization, which is also critical for understanding various diseases [27].

### 2.4. Choroidal Vascularity Index

The CVI is a recently introduced parameter, which allows for in-depth analyses of choroidal vascularization. This technique was developed owing to the major limitations of CT measurements, such as their inability to differentiate the stromal from the vascular component of the choroid, and the CT variability [34,35]. Hence, there was a need for a parameter that is less variable, more repeatable, and that allows for the study of the choroid in toto, considering both its stromal and vascular components. The quantitative parameter of the CVI was introduced by Agrawal et al., and is defined as the ratio of the luminal choroidal area (LCA) over the total choroidal area (TCA) [5]. The authors introduced this parameter with the specific intent of assessing the vascular status of the choroid, using a segmentation and binarization technique initially proposed by Sonoda et al., with some differences [5,36]. In greater detail, Sonoda et al. presented a method of assessing the subfoveal LCA and stromal choroidal area (SCA). This involved an image binarization process of the EDI SDOCT foveal scan, utilizing the freely available ImageJ software (vers. 1.5, National Institutes of Health, Bethesda, MD, USA) [36,37].

**Figure 2:**
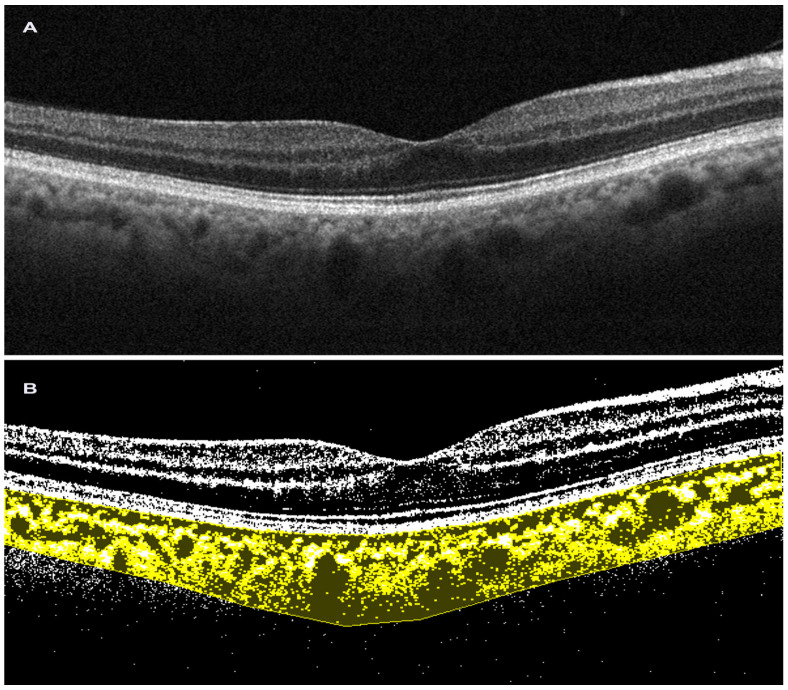
Choroidal vascularity index performed on spectral-domain optical coherence tomography scan. (**A**). Horizontal b-scan SD OCT image (Solix, Optovue Inc, Freemont, CA, USA); (**B**). Image after binarization in choroidal vascularity index processing (Image J, Fiji).

The methodology proposed by the authors involves the analysis of 1500 µm wide EDI SDOCT scans after selecting a region of interest (ROI) corresponding to the choroid, with its upper margin at the RPE level and the lower margin at the choroid–scleral interface (CSI). Three choroidal vessels with a diameter greater than 100 µm are randomly selected and the average reflectivity of these areas is calculated by the software. Then, using Niblack’s autolocal threshold tool, the image is binarized to obtain a clear view of the CSI. The image is then converted into RGB (red, green, and blue) colors to allow the color threshold tool to select dark pixels. Then, the TCA and area of dark pixels, which is the LCA, are calculated [36,37] (Figure 2). Agrawal et al., introduced some innovations to the CVI measurement technique and proposed the selection of the subfoveal choroidal area only after the image was binarized, in order to obtain a better definition of the CSI and a more precise image selection [5].

As the choroid is primarily a vascular structure, the characterization of this novel vascular index may help to further clarify the role of the vascular processes within the choroid and assess the development and progression of disease [38]. Since its introduction, many studies have sought to characterize the baseline values in the healthy choroid. Agrawal et al., in 2016, estimated that a normal CVI was about 65.6 ± 2.3% in healthy individuals, suggesting that vascular tissue represents approximately two thirds of normal subfoveal choroidal volume [5]. The CVI is considered to be a more stable choroidal assessment index than CT, because it is not affected by several physiological factors, such as intraocular pressure (IOP), axial length, refraction, and age [39,40]. Ruiz-Medrano et al. found that the CVI is higher under 18 years of age; they justified this result by observing that the LCA is higher in young people, decreasing with aging, while the SCA remains stable [41]. Diurnal changes in the CVI in healthy individuals were studied in an observational study with a small sample, which found no significant variation in the subfoveal CVI during daytime [42]. Repeatable results have also been obtained from groups calculating the CVI in various ocular and systemic vision-threatening diseases [43]. An additional plus for CVI accuracy and repeatability is that no significant instrument-related differences are found (swept-source vs. spectral-domain), but it is dependent on the SDOCT image quality, because if the CSI cannot be defined, then the CVI is unreliable [44]. In this regard, a shadow compensation visualization technique is suggested in order to remove the retinal blood vessels projection shadows that could interfere with the choroid visualization during SDOCT imaging. Various techniques have been suggested, but the description of these goes beyond the subject of this review and more data are required to validate these approaches [45]. Overall, the advantages of the CVI parameter are its stability and reduced variability compared to CT, being influenced by fewer physiological factors [40]. Multiple studies have evaluated its effectiveness as a tool for evaluating the prognosis and progression of eye and systemic diseases, with promising results [46,47]. To date, a limitation is that the CVI is used only for research purposes, as it is a parameter that requires long calculation times using specific software, thus constituting a restriction in clinical practice.

### 2.5. Optical Coherence Tomography Angiography

OCTA technology is based on the concept of motion detection. Basically, vascular flow is identified using the movement of erythrocytes as an intrinsic contrast agent. Therefore, no intravenous dye is used [48]. In brief, B-scan images are acquired in rapid succession and built-in OCTA software enables motion detection based on a differential analysis of the sequential scans. The movement of flow or blood cells creates contrast, and the motion contrast is measured by the emission of a decorrelation signal, which appears on the OCTA scan as white. Multiple B-scans are automatically performed at the same site, and the structural images are compared pixel by pixel to detect signal changes that occur due to erythrocyte sliding. The alterations observed between consecutive B-scans are represented as a motion contrast image. These B-scans can be compared either in pairs or through various combinations employing diverse algorithms [48,49]. OCTA produces three-dimensional flow images that require appropriate segmentation to evaluate vascular abnormalities. This segmentation is performed by built-in instrumental software, which selects reference planes or surfaces. In healthy eyes, the segmentation algorithm determines these layers very accurately. In contrast, in pathological conditions, where there are retinal abnormalities, it is often necessary to perform manual segmentation to correct for errors. OCTA allows for the segmentation of choroid-retinal vascularization into slabs: the superficial capillary plexus, the deep capillary plexus, the outer retina, and the CC [50].

To obtain volumetric OCTA data covering a specific area of the retina, repeated B-scans are conducted at various points using a raster scan pattern. The resulting OCTA volume allows for a three-dimensional visualization of the retinal microvasculature, and this is commonly presented by segmenting different retinal layers and displaying an en-face view similar to dye-based imaging techniques such as fluorescein angiography (FA) or indocyanine green angiography (ICGA) [48]. The most dependable approach for visualizing and interpreting OCTA images involves assessing both the en-face OCTA image and the B-scan with flux overlay, alongside the corresponding en-face structural OCT image [51]. The introduction of this new imaging technique has enabled the study of the CC without the need for invasive imaging techniques that require the use of dye, such as ICGA.

**Figure 3:**
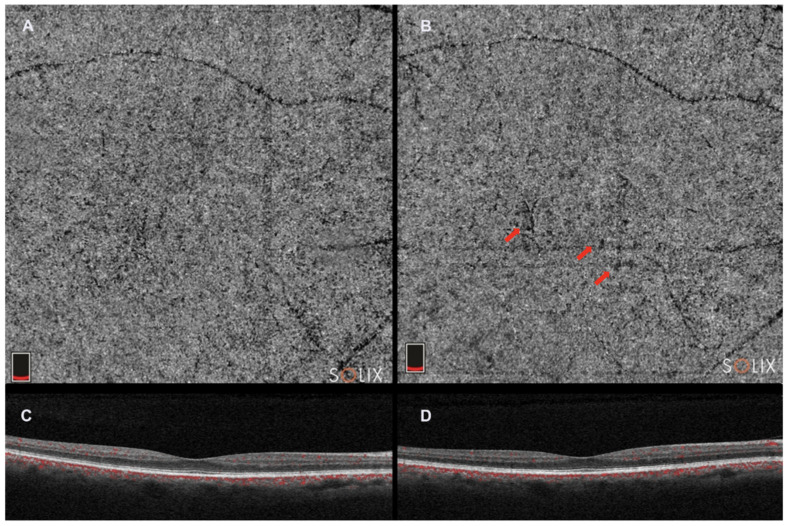
Optical coherence tomography angiography (Solix, Optovue Inc., Freemont, CA, USA). (**A**). choriocapillaris slab; (**C**). horizontal b-scan image; and (**B**,**D**). choriocapillaris slab and horizontal b-scan image in the same patient 2 years after. Red arrows indicate choriocapillaris flow deficits in the choricocapillaris angio slab.

OCTA demonstrates a lateral resolution similar to structural SDOCT and can identify CC blood flow by creating contrast between the RPE and CC [52,53,54,55]. This flow signal generates a distinctive image of the CC, showing a granular pattern with light and dark areas of varying sizes (Figure 3). The dark areas indicate relative decreases in the local flow signal and are referred to as signal voids [56]. Analyzing the number and size of these signal voids establishes a relationship linked to the CC flow, which may undergo changes during aging, disease, and potentially in the progression to late stages of AMD [57].

The interscan time is a crucial factor in OCTA motion contrast detection [58,59,60]. When other parameters remain constant, longer interscan times enhance motion sensitivity as more time elapses between repeated B-scans. However, this might lead to increased mass eye movements that can overpower the blood flow signal [61]. On the other hand, shorter interscan times reduce motion sensitivity, but also minimize the adverse impact of mass eye movements. Additionally, shorter interscan times are more effective in detecting flow impairment [48]. Commercial instruments have interscan times of 4–5 ms, while high-speed research instruments may have interscan times of 1.5 ms or less.

Another limitation of OCTA is its reliance on motion contrast to visualize the microvasculature, necessitating multiple re-scans of the same retinal location in its imaging protocols. As a result, OCTA demands higher imaging speeds or longer acquisition times compared to structural SDOCT. Moreover, OCTA lacks the ability to assess permeability changes or vascular leakage, which are commonly visualized through FA or ICGA. The appearance of OCTA image data is heavily influenced by various factors, including the specifics of the SDOCT instrument, scanning protocols, signal processing, and methods employed to derive the OCTA information from the structural SDOCT data. Algorithms can vary significantly between different types of OCT instruments. Therefore, particular care should be taken when comparing the results between different instruments. Finally, OCTA images may have many more types of artifacts than structural images and thus may be vulnerable to misinterpretation [49,62,63].

## 3. Test Results for Age-Related Macular Degeneration

AMD affects about 200 million people worldwide, and by 2040, this number is projected to rise to close to 300 million [64]. In Europe, AMD has a prevalence in subjects older than 60 years of 25.3% for early AMD forms and a prevalence of 2.4% for late AMD forms [65]. The prevalence of these various forms of AMD can also vary in relation to race and ethnicity. In fact, a recent prevalence study conducted in the United States showed a lower prevalence of early AMD in non-Hispanic black individuals (7.16%) than in non-Hispanic white individuals and Hispanic individuals (12.30% and 12.17%, respectively). In addition, the authors showed a lower prevalence of late AMD in Hispanic individuals and non-Hispanic black individuals (respectively 0.38% and 0.65%) compared to non-Hispanic white individuals (1.03%) [66]. In the Asian population, the prevalence rate is similar to that of Europeans, even if there is a major rate of a particular form of exudative AMD, such as polypoidal choroid vasculopathy (PCV) [67]. AMD is a multifactorial disease and its major risk factor is age, although constitutional, environmental, and genetic risk factors have been identified. Among the latter, cigarette smoking, an increased body mass index, hypertension, hyperlipidemia, and low omega-3 fatty acid intake have been recognized as risk factors [68]. AMD can be classified into early and late stage [69]. The early stage implies changes in the RPE that clinically, upon ophthalmologic examination, appear as areas of abnormal pigmentation in the macular area. In addition, there is the formation of drusen, which are deposits of amorphous material with a lipid content localized below the RPE. They represent the slowing down of the metabolic activity of RPE cells and, upon fundus examination, appear as small yellowish nodular formations [70,71]. A particular form of drusen are subretinal drusenoid deposits (SDD), also known as reticular pseudodrusen, which are formations very similar to drusen but located above the RPE, that are characterized by a higher risk of progression through geographic atrophy or choroidal neovascularization [72,73].

The late stage of AMD can be distinguished into an atrophic (or dry) form and an exudative (or neovascular) form. The atrophic form accounts for 80–90% of late AMD and is characterized by atrophic alterations of the photoreceptors, RPE, and CC. It shows a slow evolution with a gradual decrease in vision [74]. The exudative form is characterized by the growth of neo-formed vessels. Based on the origin of these vessels, macular neovascularization (MNV) is distinguished into three types: MNV type I, when the vessels originate from the CC and grow under the RPE; MNV type II, when the vessels grow between the RPE and the neuroepithelium; and MNV type III, when the vessels originate grow in neuroepithelium and deepen and anastomose with the choroid [75]. In addition to these types, exudative AMD also includes polypoidal choroid vasculopathy (PCV), characterized by subretinal polypoidal vascular lesions associated with a serous detachment of the RPE, particularly common in African Americans and Asian populations [76].

AMD pathogenesis involves changes in the RPE and Bruch’s membrane that are maintained by choroidal blood vessels; thus, choroidal vascular alterations may be associated with AMD [77,78]. Hence, it is important to analyze the choroidal vasculature and its alterations in AMD [79,80]. Histologically, alterations in the choroidal interstitial stroma have been observed in eyes affected by AMD [81]. This provides evidence supporting the involvement of impaired choroidal perfusion in the disease’s development [82]. Biesemeier et al. and Li et al. have put forth a hypothesis suggesting that the initial damage in AMD patients may manifest at choroidal level. Specifically, they reported that CC impairment precedes RPE degeneration in AMD patients, indicating that AMD is a vascular disease directly influenced by the choroid [83,84]. Considerable interest has developed in the analysis of the choroid in the pathogenesis of AMD, in order to identify biomarkers to predict the progression of the disease.

### 3.1. CVI in AMD

The introduction of the CVI as a biomarker of choroidal status has allowed for a more complete understanding of the pathological changes in the choroid in AMD [40]. The advantage of the CVI is that it is a more sensitive biomarker for detecting choroidal changes than CT, by including changes in both the vascular and stromal components of the choroid. Furthermore, the CVI is not associated with aging [85,86]. Several studies have reported alterations in the CVI specifically in patients with AMD or GA [87,88], and a reduction in the CVI in patients with AMD as a sign of choroidal ischemia, which is a well-known risk factor for subsequent neovascularization [89,90]. Studies performed on patients with early AMD have shown reductions in the CVI of eyes with drusen [35,91]. Abdolrahimzadeh et al. found a reduction in the CVI in patients affected by SDD and drusen with respect to healthy subjects, and the CVI was significantly reduced in the SDD eyes with respect to the eyes with conventional drusen [92]. Koh et al. and Giannaccare et al. performed studies on unilateral AMD patients, showing that affected eyes had a significantly lower CVI than fellow healthy eyes. As the authors assessed, this finding suggests that the fellow eyes may also have had a subclinical form of disease with an initial deficiency of the choroidal vasculature, possibly indicating a risk of the future development of AMD. In addition, Giannaccare et al. found that changes in the SCA were insignificant, probably due to increased stromal content [88,89]. In a recent study by Velaga et al., various choroidal parameters, including the CVI, were found to show alterations in intermediate AMD [93]. Additionally, other research studies have revealed a reduction in the CVI among GA patients, and this parameter tends to deteriorate over time. Sacconi et al. compared different cohorts of AMD, in particular patients with drusen, patients with SDD, and patients with GA, to identify different states of the vascular and stromal components of the choroid [94]. They showed a significant reduction in the CVI in the SDD and GA cohorts. In addition, a greater reduction in the CVI was found in patients with GA than in patients with only drusen and SDD. The researchers put forward a hypothesis that the decline in the CVI might have been linked to the gradual decrease in choroidal thickness (CT) across the three study cohorts [94]. Furthermore, they demonstrated that the reduction in the CVI was correlated with a decrease in the LCA, while the SCA remained relatively stable, supporting the notion of progressive choroidal vascular degeneration with stromal replacement during AMD progression [94]. On the other hand, Zhou et al. and Breher et al. reported different findings, indicating that the CVI did not exhibit significant changes associated with mean CT [85,86]. Some authors have investigated changes in the CVI in advanced exudative AMD. Invernizzi et al. conducted a study on patients with neovascular AMD, correlating changes in the CT and CVI with disease activity, conducting a type of analysis distinct in terms of the type of MNV. The authors showed a significant change in the CVI from the inactive to the active phase of the disease only in type 1 MNVs, while less noticeable changes were found in type 3 MNVs. The authors justified their results by the nature of type 1 MNV, which, being below the RPE, induces more pro-inflammatory mediators in the choroid [95]. These results were subsequently confirmed by Toto et al., who found a statistically significant reduction in the CVI in MNV type 1 patients compared to healthy controls, and in MNV type 1 patients compared to those with MNV type 2 [96].

Based on these studies, the CVI could play a prognostic role in AMD patients. Although patients may potentially benefit from close monitoring and preventive measures, further longitudinal studies conducted with the CVI are needed to evaluate this hypothesis [89].

### 3.2. OCTA in AMD

Choroid evaluation is of interest in the study of eyes with early and intermediate AMD. More specifically, several authors have reported reduced CC perfusion density in patients with drusen, SDD, and GA, prompting a crucial role of whole choroidal impairment in the pathogenesis of AMD [97,98]. Evaluations of the CC in patients with AMD can be difficult, owing to shadow artifacts due to overlying structures such as drusen and neovascularization. Therefore, it is very important to evaluate OCTA images of the CC with caution, as signal attenuation artifacts can alter the results. Consequently, many of the initial studies that analyzed the CC in early/late AMD were limited to its evaluation outside the drusen areas, rather than directly under drusen. This may be considered as an important limitation, as previous histopathological studies have suggested that drusen formation may not be random, but influenced by the anatomy of the underlying CC. Mullins and coworkers conducted an in-depth histological evaluation of the CC, revealing the existence of nonfunctioning capillary segments called phantom vessels within the CC network [99]. Their research demonstrated an interesting positive correlation between the number of phantom vessels and both age and the presence of drusen. On a related note, Curcio and colleagues also investigated the presence of phantom vessels in the eyes of elderly patients, and they additionally observed that eyes with basal linear deposits exhibited a higher proportion of these nonfunctioning capillary segments [100]. Biesemeier and colleagues found a more extensive loss of the CC in histological sections of eyes with AMD [83]. Decreased local perfusion of the CC can put the overlying RPE at risk, which can lead, in a vicious circle, to further impairment of the CC [101]. In a similar direction, late AMD can be viewed as a phase change, influenced in part by an altered CC flow, and in part by subsequent RPE distress [102]. SSOCTA systems employ a longer wavelength, provide a better penetration of the RPE, and may partially overcome the artifacts and shadowing limitations, improving the ability to study the CC in eyes with AMD [59]. Li et al., in a recent study, analyzed the CC flow in eyes with SDD using SSOCTA, where the quantification of the CC demonstrated a significant reduction in flow in eyes with SDD compared to those with drusen [101].

According to Borrelli et al., there was a notable increase in CC flow impairment specifically under and in the immediate vicinity of drusen [97]. Moreover, Nesper et al. found that eyes with SDD exhibited a greater area of CC flow impairment compared to eyes with drusen but without SDD [103]. In patients with GA, a greater impairment of CC flow around the affected area has been reported [104,105]. These results support the hypothesis that the CC may be important in the pathogenesis of AMD in the presence of SDD. Moult et al. performed a study using SSOCTA and showed that areas of GA were associated with an altered focal CC flow [106].

Numerous studies have also evaluated changes in the CC flow of patients with neovascular AMD. It is now known that there is impairment of the CC flow in MNVs type 1, with the characteristic hypoperfusion ring, “dark halo”, around the lesion, and that progressive CC ischemia contributes to the development of MNVs type 3 [107].

Interestingly, in recent years, it has been suggested that choroid study through OCTA may also play a role in assessing the response to intravitreal anti-VEGF injections. In fact, Viggiano et al. conducted an OCTA study with the purpose of evaluating changes in the vascular density of the CC in patients with type 1 MNVs, before and after injections of anti-VEGF. The authors highlighted significant differences in CC perfusion after intravitreal injection therapy, demonstrating the remodeling of CC perfusion in the area surrounding the dark halo [108]. The same results were recently confirmed by Cabral et al., who showed a recapitulation of CC areas with insufficient blood flow in type 1 MNVs, resulting in the preservation of visual acuity [109].

Although these early investigations have provided new insight into the CC flow in healthy and diseased eyes, OCTA continues to be a technology undergoing rapid development. However, artifacts resulting from segmentation errors, signal attenuation, projection, and eye movement remain a significant concern. Identifying and acknowledging these artifacts is crucial to preventing the misinterpretation of the images. Future advances in OCTA will provide wider-field images, better artifact removal, and more accurate image segmentation and analyses [48,49].

## 4. Conclusions

The CC plays a crucial role in the pathogenesis, progression, and overall course of several diseases, such as AMD. Gaining a better comprehension of this vascular layer is likely to significantly influence the overall understanding of various ocular conditions. With continuous advancements in imaging technology, new possibilities have emerged for enhanced evaluations of the CC, enabling more effective diagnosis and management in clinical practice. Significant improvements in SDOCT technology have led to sophisticated, noninvasive techniques that produce images with an excellent resolution of the retina. The newly introduced EDI technology allows for the accurate visualization and study of the choroid.

The CVI, a newly proposed parameter, holds the potential to facilitate the quantitative measurement and analysis of the choroidal vasculature. It has introduced fresh perspectives in choroidal health research by offering the ability to assess the proportions of vascular and stromal components. This more accurate understanding of choroidal vasculature alterations is particularly relevant for individuals with AMD. CVI assessments stand out as a superior and more informative approach compared to traditional parameters such as CT.

Despite the valuable insights provided in this review regarding the analysis of the choroidal vasculature in both normal and diseased eyes, it is essential to acknowledge that CVI measurements are still in their evolutionary stages. Ongoing development is taking place to refine and optimize the process. Both automated and semi-automated CVI analyses require a pristine and comprehensive visualization of the entire choroid to deliver reliable results. However, it is important to note that limitations persist due to the artifacts arising from OCT signal attenuation and projection, which may impact the accuracy and precision of CVI measurements. As research in this field continues to progress, overcoming these challenges will be paramount to unlocking the full potential of the CVI as a powerful tool in the assessment of choroidal health and its associated diseases.

Regarding OCTA, this is also a technology that is still evolving, although it has already provided new insights into the CC. Artifacts due to errors in segmentation, signal attenuation, projection, and eye movement still remain a problem. Table 1 summarizes the advantages and disadvantages of the CVI and OCTA in the study of the choroid.

In the near future, CVI analyses may be integrated into the software of all SDOCT instruments, becoming an integral tool in clinical practice; instead, future advances in OCTA will provide wider-field or montage images, a more accurate and consistent removal of artifacts, and improved image segmentation and analysis.

## Figures and Tables

**Table 1:** Advantages and disadvantages of choroidal vascularity index versus optical coherence tomography in the study of the choroid.

Parameter	Advantages	Disadvantages
CVI	- More stable and less variable parameter with respect to choroidal thickness evaluation - Allows the study of both stromal and vascular components of the choroid [34,35] - Not affected by factors such as axial length, intraocular pressure, refraction, and age [39,40] - No significant differences related to the type of instrument (swept source vs. spectral domain) [44]	- Used for research purposes only - Requires very long processing times and specific software - Affected by artifacts due to OCT signal attenuation and projection - Dependent on SDOCT image quality [44]
OCTA	- Provides three-dimensional visualization of the choroidal microvasculature - Allows the study of the choriocapillaris without the use of invasive imaging techniques - Used for routine clinical follow up owing to simple and rapid examination	- Does not provide information on changes in permeability or vascular leakage - Interscan time plays a critical role in motion sensitivity [58,59,60] - Artifacts can be caused by segmentation errors and eye movement

CVI: Choroidal vascularity index; OCT: optical coherence tomography; SDOCT: spectral domain optical coherence tomography; and OCTA: optical coherence tomography angiography.

## Data Availability

Not applicable.
